# Association of serum ferritin with non-alcoholic fatty liver disease: a meta-analysis

**DOI:** 10.1186/s12944-017-0613-4

**Published:** 2017-12-02

**Authors:** Shui-Xian Du, Lin-Lin Lu, Ning Geng, David W. Victor, Li-Zhen Chen, Cong Wang, Hai-Yan Yue, Yong-Ning Xin, Shi-Ying Xuan, Wen-wen Jin

**Affiliations:** 1 Medical College of Qingdao University, Qingdao, 266071 China; 2 Department of Gastroenterology, Qingdao Municipal Hospital, 1 Jiaozhou Road, Qingdao, Shandong Province 266011 China; 3 Digestive Disease Key Laboratory of Qingdao, Qingdao, 266071 China; 4 Central Laboratories, Qingdao Municipal Hospital, Qingdao, 266071 China; 5 College of Medicine and Pharmaceutics, Ocean University of China, Qingdao, 266003 China; 6 Department of Gastroenterology, Dalian Medical University, Qingdao, 266011 China; 7 Hepatology & Transplant Medicine, Department of Medicine, Houston Methodist Hospital, Houston, USA

**Keywords:** Non-alcoholic fatty liver disease (NAFLD), Meta-analysis, Serum ferritin (SFL), Non-alcoholic steatohepatitis (NASH), Non-alcoholic fatty liver (NAFL)

## Abstract

**Background:**

A growing number of studies reported the connection between the level of serum ferritin (SFL) and non-alcoholic fatty liver disease (NAFLD). However, such connection was still disputable. The aim of our meta-analysis was to estimate SFL between the groups as below: patients with NAFLD against control group; non-alcoholic steatohepatitis (NASH) patients against control group; non-alcoholic fatty liver (NAFL) patients against a control group and NASH patients vs NAFL patients.

**Methods:**

We screened the studies in PubMed, EMBASE, the Cochrane Database and the Cochrane Central register controlled trials from the beginning to July 10, 2016 to find the studies indicated the connection between SFL and NAFLD (NAFL and/or NASH). Fourteen published studies which evaluate the SFL in NAFLD patients were selected.

**Results:**

Higher SFL was noticed in NAFLD patients against control group (standardized mean difference [SMD] 1.01; 95% CI 0.89, 1.13), NASH patients against control group (SMD 1.21; 95% CI 1.00, 1.42), NAFL patients against control group (SMD 0.51; 95% CI 0.24, 0.79) and NASH patients against NAFL patients (SMD 0.63; 95% CI 0.52, 0.75). These results remained unaltered actually after the elimination of studies which were focused on paediatric or adolescent populations. Higher SFL was presented in NAFLD patients against the control group (SMD 1.08; 95% CI 0.95, 1.20) in adults and NASH patients against NAFL patients in adults (SMD 0.74; 95% CI 0.62, 0.87). The connection between SFL and NASH against NAFL group in paediatric or adolescent populations was observed inconsistently (SMD 0.10; 95% CI -0.18, 0.38).

**Conclusions:**

The level of SFL was elevated in patients with NAFLD (NAFL and/or NASH) compared with the controls. Compared with NAFL, The level of SFL was increased in NASH. The result remained unaltered actually after the elimination of studies focused on paediatric or adolescent populations.

## Background

Non-alcoholic fatty liver disease (NAFLD) is the most prevalent chronic liver disease worldwide. The prevalence of it was 25.24% of the overall population [1, 2]. NAFLD comprises of a wide spectrum of liver damage, including non-alcoholic fatty liver (NAFL) and non-alcoholic steatohepatitis (NASH), as well as cirrhosis and fibrosis which can be complicated by hepatocellular carcinoma and liver failure [3]. NAFLD is frequently associated with insulin resistance (IR) and metabolic syndrome (MS) and it is typically manifested as type 2 diabetes mellitus (T2DM), dyslipidemia, obesity, as well as hypertension [4]. Therefore, the diagnose of NAFLD at very early stage is necessary.

Liver biopsy is considered to be a principle procedure for the diagnosis of patients with NAFLD [1], however, it is invasive [5]. NAFLD may be recognised only after the elimination of the other liver disorders during the image evaluation [6]. There are several researches which use magnetic resonance imaging (MRI) proton density-fat fraction to diagnose NASH. It is a non-invasive method to assess and quantify hepatic steatosis in NAFLD patients [7]. Nonetheless, its precise cut-off value has not been estimated. In addition, the tackle for MRI is not widely available because it is expensive. Thus, scientists are actively looking for cheap and non-invasive biological markers which may be helpful in the diagnosis of NAFLD and the prognosis of NAFLD.

Serum ferritin (SFL) is a protein expressed in an acute phase, so its level is elevated in the case of liver necrosis, inflammation [8]. Some recent investigations stated that the level of SFL can be an irrespective indicator to assess the progression of hepatic fibrosis in the patients with NAFLD because of its association with hepatic iron storage and hepatic inflammation. Researchers came to a conclusion that SFL is higher in patients with NAFLD that might be linked with insulin resistance and hepatocyte damage [9, 10]. However, some empirical evidences showed that SFL can not indicate the stage of NAFLD [11]. These connections are still disputed.

According to our research, no previous meta-analysis had been done to estimate the connection between SFL and NAFLD (NAFL and/or NASH). The purpose of this meta-analysis was to investigate the quantitative connection between the SFL and NAFLD (NAFL and/or NASH) and to estimate the influence factors of this relationship. The other aim was to evaluate whether SFL can be treated as a potentially effective and less-invasive biological marker in patients with NAFLD (NAFL and/or NASH).

## Methods

### Literature search

According to the PRISMA directions [12], the published studies through a systematic screening of PubMed, EMBASE and Cochrane Database from the beginning to July 10, 2016 were found. Keywords for the search were as follows: (“Non-alcoholic Fatty Liver Disease”, “Non-alcoholic Fatty Liver Disease”, “NAFLD”, “Non-alcoholic Fatty Liver Disease”, “Fatty Liver”, “Non-alcoholic”, “Fatty Livers”, “Non-alcoholic”, “NASH”, “Liver”, “Non-alcoholic Fatty”, “Livers”, “Non-alcoholic Fatty”, “Non-alcoholic Fatty Liver”, “Non-alcoholic Fatty Liver”, “Non-alcoholicSteatohepatitis”, “Non-alcoholicSteatohepatitides”, “Steatohepatitides”, “Non-alcoholic”, “Steatohepatitis”, “Non-alcoholic”) and (“Iron”,“Ferritin”).

### Inclusion criteria

Two authors (DU SX and LU LL) irrespectively screened the suitable records studies: 1)are published in English; 2) the original observations including population of any sex or ethnicity; 3) provide input related to SFL and NAFLD (NAFL and/or NASH); 4) include the comparison of SFL between NAFLD (NAFL and/or NASH) patients and controls; 5) include the comparison of SFL between NAFL and NASH patients as well.

### Data extraction

Two researchers (DU SX and LU LL) irrespectively elicited the data from particular eligible articles. Recorded input consisted of: first author’s name, venue of study, year of publication, design of study, number of patients as well as controls and their gender, the histological degree of NAFLD (if provided), method of NAFLD assessment, additional information, mean values and standard deviation (SD) of SFL.

### Quality assessment

The methodological value of studies was estimated by the NOS (Ottawa Hospital Research Institute, Ottawa, ON, Canada) [13] by two reviewers (DU SX and XIN YN) who involved in our study.

### Categories of NAFLD

In accordance to the benchmarks of NAFLD activity score (NAS). NAS of >5 correlated with a definition of NASH and NAS < 5 was defined as NAFL [14].

### Outcomes

The primary result of this meta-analysis was the standardise mean difference (SMD) of SFL among NAFLD patients and control groups. NAFLD patients were categorised as NAFL or NASH based on NOS [14]. Afterwards, we performed a comparison of SFL among the following groups: [1] NAFLD patients against control group; [2] NAFL patients against control group; [3] NASH patients against a control group and [4] NASH patients against NAFL patients.

### Statistical analysis

Our meta-analysis used SFL as basic result. SFL was described as the standard mean difference (SMD) displaying 95% confidence intervals (CI). The variety of the statistical results were estimated by the Cochran Q test and the I^2^ statistic. Heterogeneity was recognised as significant when the Cochran Q test was *p* < 0.05 or I^2^ was more than 50% [15, 16]. Depending on the absence or presence of heterogeneity, different types of models including fixed-effects and random model were used. All subgroups were subjected to analyse. We investigated all related articles on the SFL individually of different types of studies (including case-control studies, prospective studies and cross-sectional). In order to explore if the level of SFL can effect the progression of NAFLD, we also investigated the SFL among NAFL patients compared with NASH patients separately.

Furthermore, we increased a sensitivity analysis through the elimination of studies focused on adolescent/paediatric population. Next, the impact of each study on the pooled measures was evaluated by ignoring one in each turn and then the summarised SMDs of the rest subjects were calculated [3]. We used Funnel plots to estimate the publication bias at first [17] and later this bias was corroborated by using Begg’s [18] and Egger’s tests [19]. Our meta-analysis was performed using Stata Statistical Software (ver. 12.0; StataCorp LP, College Station, TX).

## Results

### Literature search

**Fig. 1:**
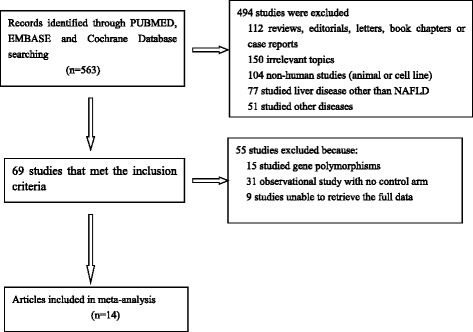
Flowchart showing the process of literature’s selection, in accordance with the PRISMA declaration

Figure 1 presents the selection process of the studies and literature search results in this meta-analysis. After the initial search, we obtained 563 results. We screened titles and abstracts, 494 of them were excluded due to plenty of reasons, including lack of primary data (reviews and meta-analysis), inappropriate topics, non-human studies, negligible population (alcoholic fatty liver disorder) and liver disease other than NAFLD. At last, 14 studies in total were chosen for further analysis after reviewing full texts

### Characteristics of the included studies

The major features of these trials were summarised in Table 1. After the whole presented workflow, 14 studies were admitted to our meta-analysis [9–11, 20–30]. Input for NAFLD patients was carried only when a control group was not included in the study (i.e. in the situation when there was a comparison of SFL only between NAFL and NASH patients). Therefore, it was impossible to compare NAFLD patients. Five studies were performed in Europe, five in Asia and four in North America. Studies in the meta-analysis included one cross-sectional study, nine case-control studies and four prospective studies. NAFLD (NAFL and/or NASH) was confirmed by hepatic ultrasonography in two studies and liver biopsy in twelve studies. The outcome measure of each study was presented in Table 2. Two studies consisted of all groups (controls, NAFL, NASH patients) [23, 25]. Three studies compared SFL between NASH patients and controls [23–25]. Two studies compared SFL between NAFL patients and controls [23, 25]. Three studies compared SFL between NAFLD patients and controls, but they didn’t carry independent evidence on both NAFL and NASH [9, 21, 28]. Ten studies compared SFL between NAFL and NASH patients [10, 11, 20, 22, 23, 25–27, 29, 30] (Table 2).

**Table 1:** Characteristics of studies contained in the meta-analyses

Study, year	Country	Number[Male/Female, mean age(years)]		Study design	Categories of NAFLD [Male/Female]		Definition	Additional information	NOS(0–9)
		case	Control		NAFL(number)	NASH(number)			
Natasha Chandok, 2011	Canada	88	NA	prospective study	60 (37/23)	28 (13/15)	liver biopsy	NA	6
Kikuko Hotta, 2010	Japan	253 (122/131,51)	578 (182/396,47)	case-control	64 (23/41,51)	189 (99/90,51)	liver biopsy.	NA	6
T-J Hsiao, 2004	Taiwan, China	43 (20/23,33)	167 (27/140,36)	case-control	NA	NA	Ultrasonography	obese	7
George BB Goh 2015	USA	405 (179/226,48)	NA	prospective study	114 (52/62,46)	291 (127/164,49)	liver biopsy.	NA	6
Michelino Di Rosa, 2013	Italy	200 (92/108)	100 (48/52,54.2)	case–control	90 (43/47,49)	110 (49/61,53)	Liver biopsy	NA	7
Ali Sazci,2008	Turkish	57 (31/26,44)	245 (106/139,45)	Case-control	NA	57 (31/26,44)	liver biopsy	NA	6
Youzhao Jiang, 2014	China	446 (351/95,46)	531 (201/330,42)	cross-sectional	NA	NA	liver biopsy.	NA	6
Hiroyuki Tsuchiya,2010	Japan	28	8 (30)	Case-control	17 (46)	11 (50)	liver biopsy	NA	6
Masato Yoneda, 2010	Japan	86	20	Case-control	24 (48)	62 (52)	liver biopsy.	NA	5
Zeljko Puljiz, 2010	Croatia	50 (32/18,43)	NA	prospective study	35	15	liver biopsy.	NA	5
B.Canbakan, 2007	US	105 (54/51)	NA	prospective study	38 (17/21)	67 (37/30)	liver biopsy	NA	4
Demircioğlu F,2014	Turkey	30 (19/11,12)	50(17/33,12)	case–control	NA	NA	ultrasonography	obese	6
Nobili V, 2013	Italy	100 (68/32,11)	NA	case–control	70 (51/19,11)	30 (17/13,11)	liver biopsy.	NA	5
Alkhouri N, 2015	USA	117 (78/39,12)	NA	case–control	49 (30/19,12)	68 (48/20,13)	liver biopsy.	NA	5

*NAFL* non-alcoholic fatty liver, *NASH* non-alcoholic steatohepatitis, *NA* not available

**Table 2:** Comparison of groups among 14 studies and after the elimination of paediatric/adolescent studies

Comparison	All studies	Excluding paediatric/adolescent studies
NAFLD vs control	1.01 (0.89,1.13)	1.06 (0.95,1.20)
*p* value	<0.0001	0.099
NAFL vs control	0.51 (0.24,0.79)	NA
*p* value	0.628	NA
NASH vs control	1.21 (1.00,1.42)	NA
*p* value	0.005	NA
NASH vs NAFL	0.63 (0.52,0.75)	0.74 (0.62,0.87)
*p* value	<0.0001	<0.0001

Data are presented as SMD (95% CI)

*NA* not available

Following comparative data were provided: three studies, NAFLD patients (*n* = 519) against control group (*n* = 748), two studies, NAFL patients (*n* = 107) against control group (*n* = 108), three studies, NASH patients (*n* = 178) against control group (*n* = 198) and ten studies, NAFL (*n* = 561) against NASH patients (*n* = 871).

### Quality of included studies

In accordance to NOS, Table 1 shows the value of included studies. Two studies scored 7, seven studies scored 6, four studies scored 5 and one study scored 4 (mean ± SD 6.15 ± 0.97). No study was eliminated due to the low NOS (score ≤ 2).

### Outcomes

**Fig. 2:**
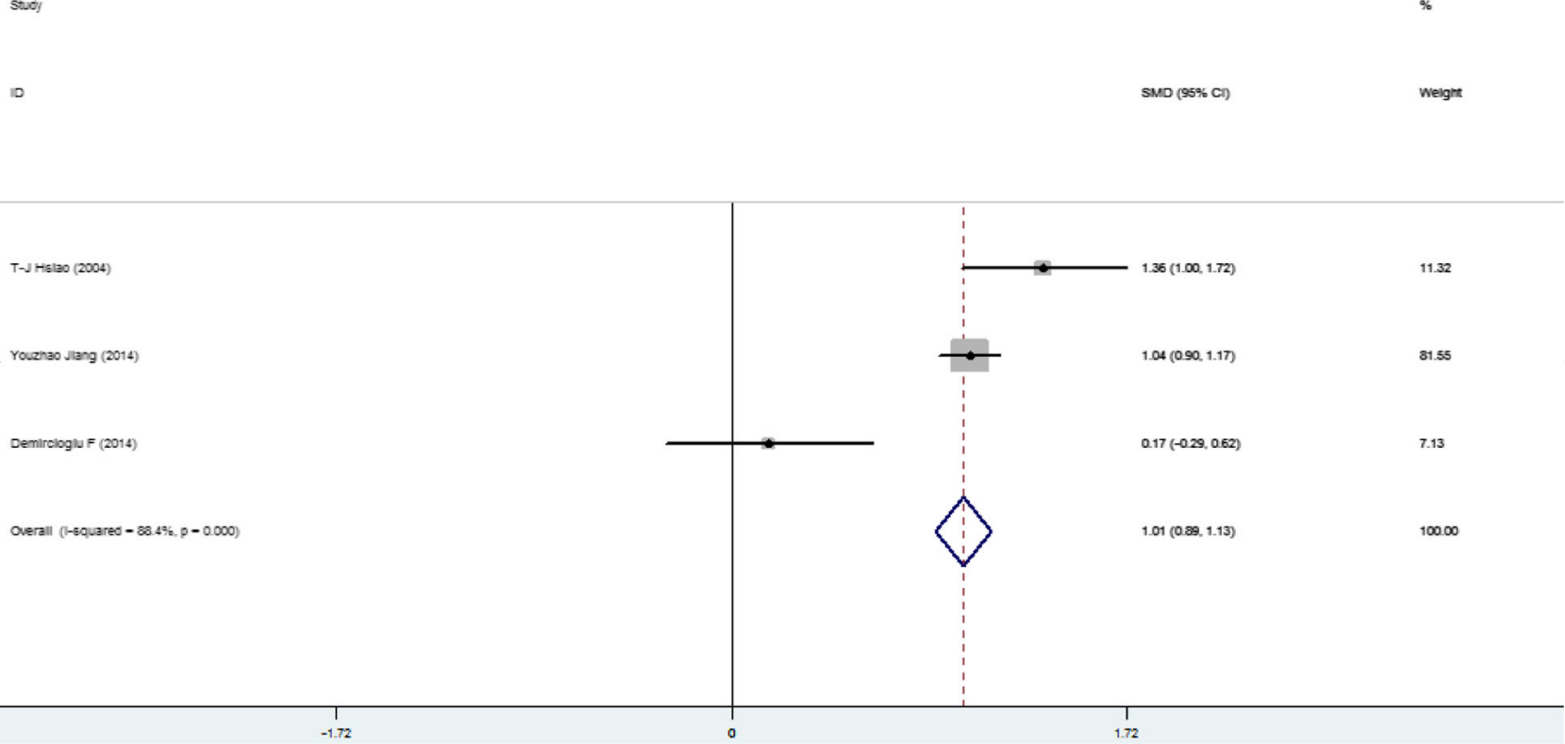
Forest plots show the juxtaposition of SFL among the groups included in the studies: NAFLD patients against control group

**Fig. 3:**
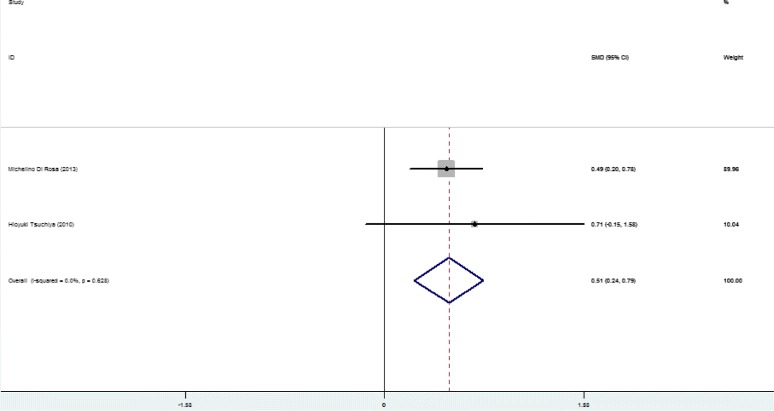
Forest plots show the juxtaposition of SFL among the groups included in the studies: NAFL patients against control group

**Fig. 4:**
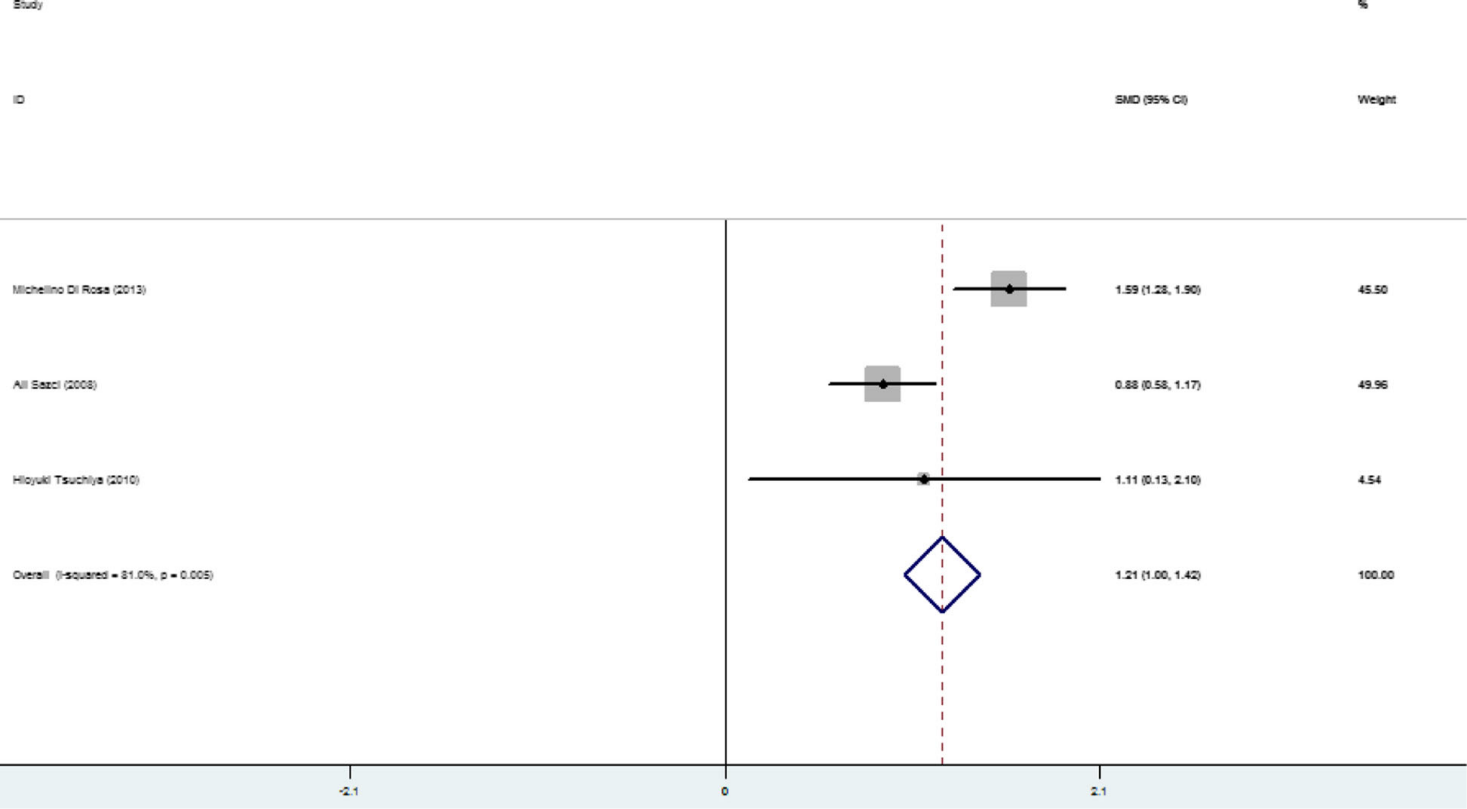
Forest plots show the juxtaposition of SFL among the groups included in the studies: NASH patients against control group

**Fig. 5:**
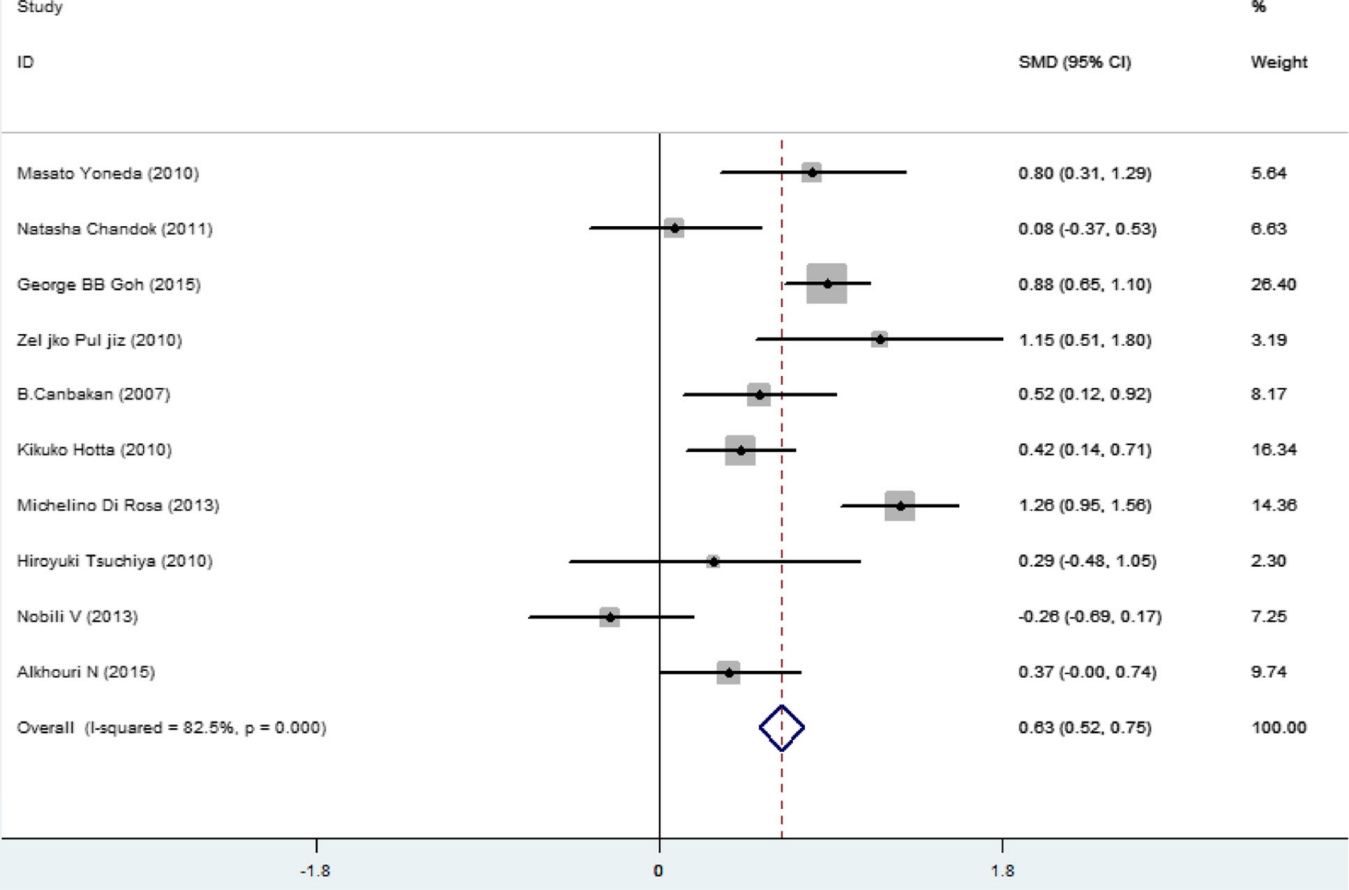
Forest plots show the juxtaposition of SFL among the groups included in the studies: NASH patients against NAFL patients

**Fig. 6:**
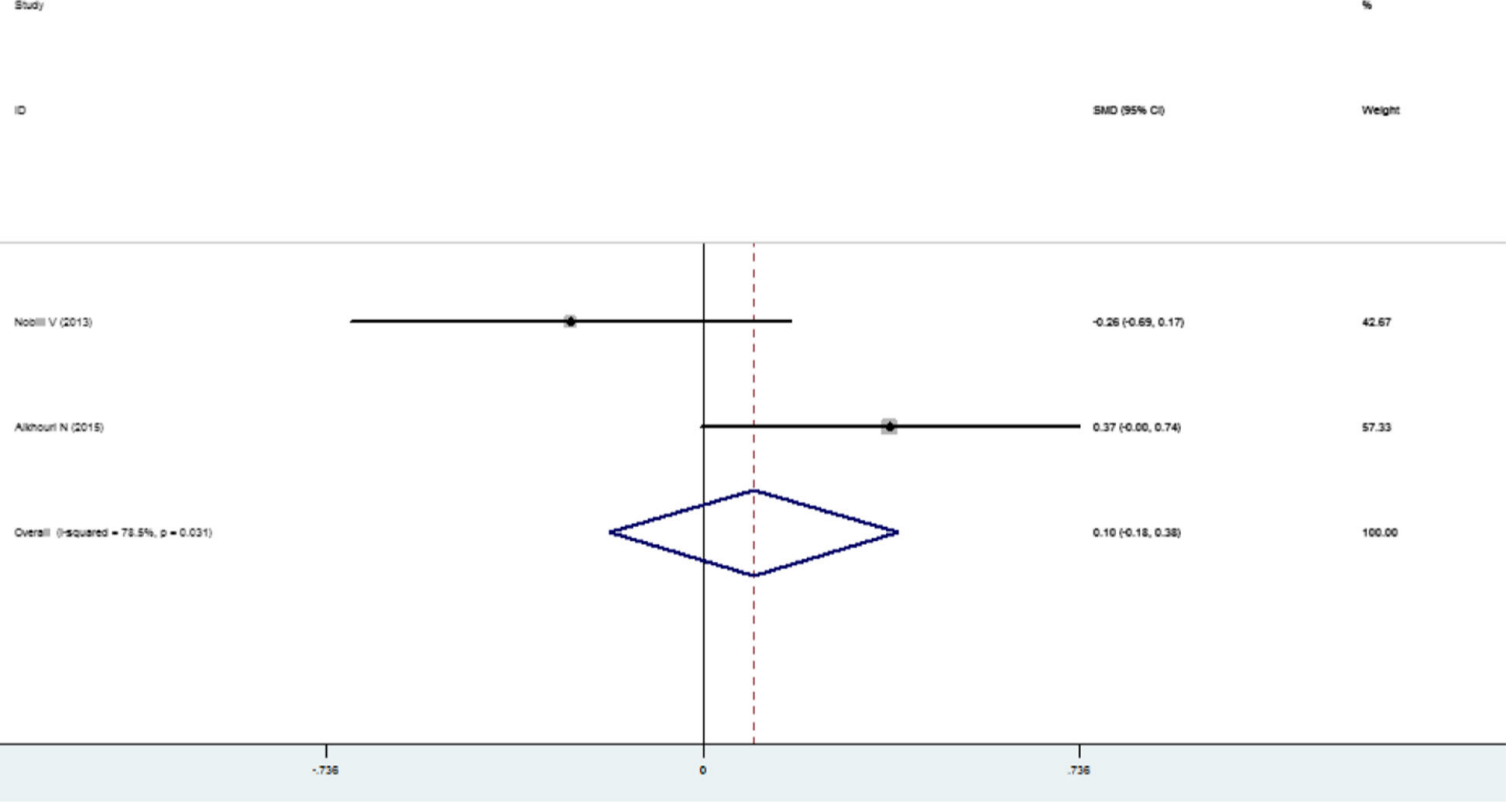
Forest plots show the juxtaposition of SFL among the groups included in the studies: NASH patients against NAFL patients in paediatric/adolescent studies

Higher SFL was noticed in the following groups: (1) NAFLD patients against controls; (2) NAFL patients against control; (3) NASH patients against control and (4) NASH against NAFL patients (Table 2; Figs. 2, 3, 4, 5 and 6). The variety amongst the studies was mild-to-severe in the case of all juxtapositions (I^2^ ranged from 0% to 88.4%; Fig. 2). There was no meaningful bias in any collation (*p* > 0.05 for all comparisons; Table 3 and Fig. 1).

**Table 3:** The analysis of publication bias in the included studies and after the elimination of paediatric/adolescent studies

Comparison	All studies	Excluding paediatric/adolescent studies
NAFLD vs control	0.602	0.317
NAFL vs control	0.317	NA
NASH vs control	0.602	NA
NASH vs NAFL	0.788	0.621

Data are showed as *p* values derived from Egger’s regression. If *p* > 0.05, there is no publication bias

**Fig. 7:**
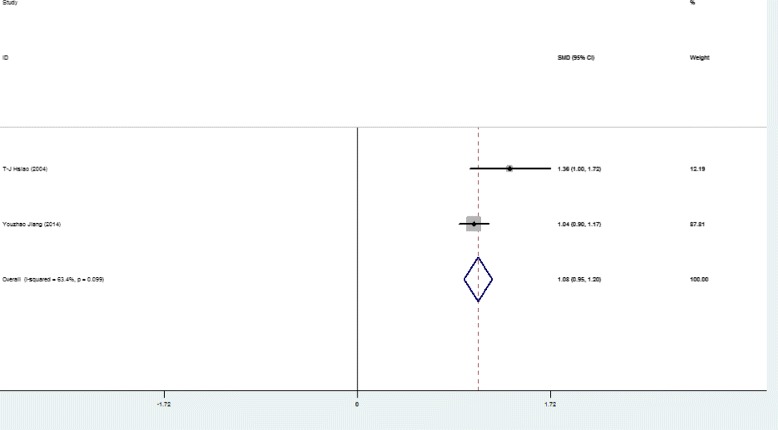
Forest plots show the comparison of SFL between following groups in the studies after the elimination of paediatric/adolescent studies. NAFLD patients against control group

**Fig. 8:**
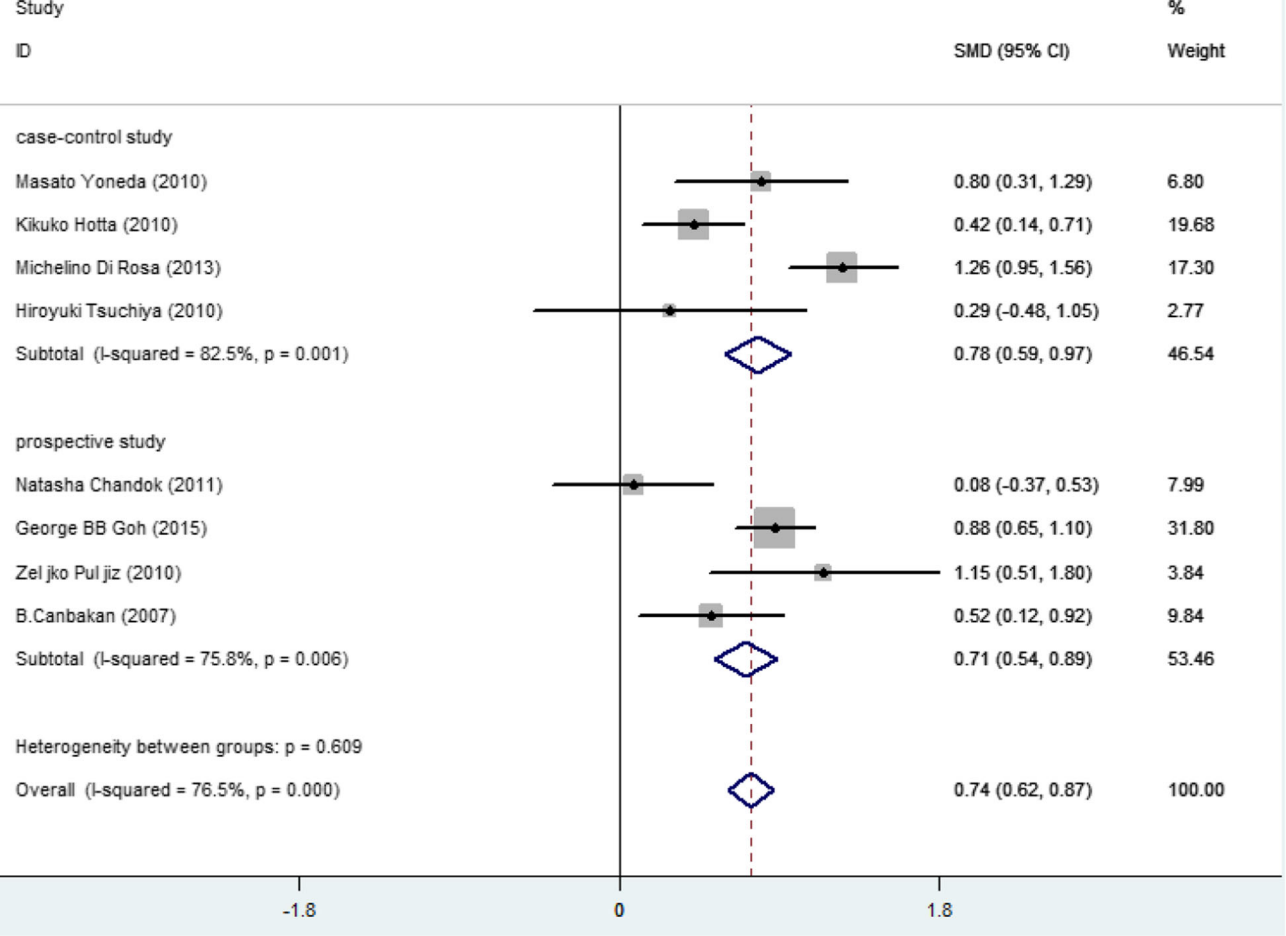
Forest plots show the comparison of SFL between following groups in the studies after the elimination of paediatric/adolescent studies. NAFL patients against control group

In the sensitivity analysis, after the elimination of paediatric/adolescent studies, there were only little alterations among groups (Figs. 7 and 8, Table 2). The estimated heterogeneity of NAFLD and control group was 63.4% and the heterogeneity in NAFL and NASH group was still 76.5%. Based on the different types of studies, subgroup showed that NASH patients showed 0.78 ng/mL higher level of SFL compared with NAFL (95% CI: 0.59, 0.97 ng/mL) (I^2^ = 82.5%, *p* < 0.001) in four case-control studies [10, 20, 23, 26], while the SMD of SFL was 0.71 ng/mL (95% CI, 0.54, 0.89 ng/mL) (I^2^ = 75.8%, *p* = 0.006) in four prospective studies [11, 22, 25, 27] (Figs. 7 and 8) after the elimination of paediatric/adolescent studies. The signs of publication bias were not observed (*p* > 0.05 for all comparisons, Table 3).

## Discussion

After performing this meta-analysis, we concluded that higher SFLcan be linked with the severity of NAFLD since the controls showed lower SFL compared with NAFL, NASH or NAFLD patients and NAFL patients showed lower SFL compared with NASH patients. The sensitivity analyses and subgroup analyses did not essentially influence or alter these conclusions. As such, SFL can be as a less-invasive and effective biological marker to prognosticate the progression of NAFLD.

In terms of the hypothetical mechanisms linking SFL and NAFLD, SFL displayed strong biological plausibility, thus it can be used as a marker in the determination of NAFLD. Existing two-hit theory which takes the progression to NASH and fibrosis into account, is the most common mechanism regarding the pathogenesis of NAFLD [31]. In this assumption, the first “hit” is IR which related with visceral obesity, resulting in free fatty acids and elevated circulating hepatic steatosis. On the other hand, the second “hit” might be induced by the additional factors which may result in inflammation of the liver and elevated oxidative stress and ultimately lead to tissue injury, steatohepatitis and fibrosis [32]. Few researches indicated that the elevated deposition of iron was an important factor in catalysing the production of reactive oxygen species through the Fenton reaction, which was suggested to be the second hit. Besides the production of reactive oxygen species [32], iron may play a role in a number of different disastrous pathways, including changed insulin signalling and lipid metabolism. In the liver, where the majority of extra body iron is retained. SFL is the main iron-storage protein. It can be increased secondary due to the steatohepatitis, obesity, histiocytic neoplasm, chronic consumption of alcohol as well as chronic inflammation including viral hepatitis [33]. Together with the elevated level of ferritin concentration, the risk of serious liver disease is increasing constantly. Manousou P, et al. [9] reported that the elevated SFL may reflect the occurrence of hepatitic failure and metabolic syndrome because of the activation of inflammatory cytokines in NAFLD patients. What’s more, Nelson JE, et al. reported that hepatic iron accumulation is correlated with hepatic fibrosis in NAFLD subjects, what is confirmed in a large number of studies focused on the pathophysiological point of view [34]. Valenti L, et al. reported that the accumulation of hepatic iron may contribute to the production of inflammatory cytokines, what might lead to the hepatic fibrosis [35]. According to the research performed by Kowdley et al. [33], the histological characteristics, including fibrosis of NAFLD, steatosis and hepatocellular ballooning,were more serious in the case of patients with higher SFL. They reported that SFL may be linked with the aggravated histological function and hepatic iron exemption among patients with NAFLD.

There are many benefits resulting from the presented study. As we know, this is the first meta-analysis which evaluates the connection between the SFL and NAFLD based on the extensive search. As NAFLD consists of a wide spectrum of disorders, our meta-analysis was carried out in order to uncover changed SFL in NAFL and NASH, comparing to the healthy controls. In addition, we also performed the evaluation of NAFL and NASH patients in order to examine whether SFL was related with the severity of NAFLD. On the other hand, the analysis was revealed the connection between SFL and NAFLD in adults and paediatric or adolescent populations separately.

However, there are some significant restraints concerning this meta-analysis. First, the majority of original studies did not match the potential confounders, such as hyperlipidemia, IR, liver enzymes and body mass index. We did not manage to confirm that SFL poses an independent risk factor for NAFLD. Second, the evaluation of liver enzymes was relatively insensitive to detect NAFLD, what may be the result of possible wrong categorization of patients with NAFLD as unaffected controls. Third, the veracity of the results was restrained due to the variety of between-study, which should be exclusively commentated in the reference to dissimilarities of BMI between compared groups. Four, we eliminated unpublished studies or abstracts from conferences, which may lead to the bias. However, such elimination is crucial in order to refrain the low-quality input, because its value cannot be evaluated in total [36]. Five, because of the lack of corroborated quality assessment instrument for cross-sectional studies, NOS, the most prevalent ratio for observational studies was used in order to eliminate low-value studies. Six, since only four studies specified that the controls constitute the same pool as the subjects do, the number of valuable case-control studies’ might also be restrained. Seven, we did not evaluate SFL in case of inflammation, fibrosis stage or steatosis individually because of the lack of the available histological lesions data. It was significantly limited by the division of groups and different histological interpretations. Finally, we did not manage to perform subgroup and sensitivity analyses in order to reveal the effects of other potential factors, such as the definition of NAFLD, gender and race, and the way of testing the SFL, due to an inadequate number of data.

## Conclusions

This meta-analysis explored that NAFLD patients showed a higher SFL, what can be related with the severity of NAFLD. These results are consistent with the hypothesis that the elevated SFL is related with IR and hepatocyte damage and it also plays a fibrotic and pro-inflammatory role during the progression of the disease. The further studies also be needed to reveal the causal role of SFL in the progression of NAFLD and the mechanism of the pathogenesis of NAFLD.

## Abbreviations

MRI: Magnetic resonance imaging
MS: Metabolic syndrome
NAFL: Non-alcoholic fatty liver
NAFLD: Non-alcoholic fatty liver disease
NASH: Non-alcoholic steatohepatitis
SFL: Serum ferritin
T2DM: Type 2 diabetes mellitus
